# Autologous stem cell transplantation with thiotepa, busulfan, and cyclophosphamide conditioning in patients with central nervous system lymphoma: a phase II study

**DOI:** 10.1007/s00277-025-06405-y

**Published:** 2025-06-14

**Authors:** Dong Hyun Kim, Taekeun Park, Junshik Hong, Dong-Yeop Shin, Inho Kim, Sung-Soo Yoon, Ja Min Byun, Youngil Koh

**Affiliations:** 1 Department of Internal Medicine, Seoul National University Hospital, Seoul, Republic of Korea; 2 Department of Translational Medicine, Seoul National University College of Medicine, Seoul, Korea; 3 Cancer Research Institute, Seoul National University College of Medicine, Seoul, Korea; 4 Biomedical Research Institute, Seoul National University Hospital, Seoul, Korea

**Keywords:** Central nervous system lymphoma, Autologous hematopoietic stem cell transplantation, Thiotepa/busulfan/cyclophosphamide conditioning, Clinical trial.

## Abstract

The prognosis of central nervous system lymphoma (CNSL) remains poor, and attempts have been made to improve outcomes through autologous stem cell transplantation (ASCT) with thiotepa-based conditioning. We aimed to assess the outcomes of thiotepa/busulfan/cyclophosphamide (TBC) followed by ASCT in CNSL. An investigator-initiated, single-arm, phase II trial was conducted to evaluate the efficacy and safety of TBC/ASCT (NCT06625359). The conditioning dose was adjusted based on age and performance status (9-day or 8-day regimen). As this trial was terminated early after enrolling 17 patients, a retrospective cohort of CNSL treated with the same protocol was included. In total, 44 patients were included in the study and classified into 8-day and 9-day groups according to the TBC regimen received. In total, 25 patients (56.8%) had primary CNSL, and 19 patients (43.2%) received the 9-day regimens. Following ASCT, 33 patients (75.0%) achieved an objective response (32 complete response and 1 partial response). The 3-year progression-free survival (46.5% vs. 52.6%, *P* = 0.49) and overall survival (60.5% vs. 73.7%, *P* = 0.77) for 8-day and 9-day groups were comparable. The 1-year cumulative incidence of non-relapse mortality in the 8-day group showed a trend toward decrease compared to that in the 9-day group (4.6% vs. 21.1%, *P* = 0.073). Our study demonstrates the efficacy of TBC/ASCT in CNSL in the Asian population. The conventional busulfan dose (9-day regimen) may be associated with higher toxicity, suggesting the potential need for a modified TBC regimen in Asian populations. Further studies are needed to identify the optimal thiotepa-based conditioning in CNSL.

**Supplementary Information:**

The online version contains supplementary material available at 10.1007/s00277-025-06405-y.

## Introduction

The involvement of central nervous system (CNS) by non-Hodgkin lymphoma is associated with an unfavorable prognosis [1, 2]. For both of primary CNS lymphoma (PCNSL) and secondary CNS lymphoma (SCNSL), high-dose methotrexate-based chemotherapy and combined modalities have improved response rates and overall outcomes [3–6]. Nevertheless, relapse rates remained high, and only a subset of patients achieved long-term remission. Consolidation therapy with whole-brain radiotherapy (WBRT) has been shown to improve disease-free and overall survival (OS) rates, but it is associated with severe neurotoxicity [7].

To overcome this dismal outcome, high-dose chemotherapy followed by autologous stem cell transplantation (ASCT) has been explored as first-line consolidation therapy or in the relapsed/refractory setting for CNS lymphoma [8–11]. Thiotepa-based conditioning regimens are commonly employed in combination with either busulfan/cyclophosphamide (TBC) or BCNU (TT-BCNU) [12–15]. TBC has demonstrated advantages in disease control compared to other thiotepa-based regimens [16]. However, high-dose chemotherapy is associated with significant toxicity, making it intolerable for some patients; in particular, the use of the TBC regimen in patients older than 60 years was associated with an increased risk of transplantation-related mortality [17]. The rarity and poor prognosis of this disease present challenges in conducting large-scale phase III trials and long-term follow-up studies in this setting.

To address this issue, we conducted a phase II study to evaluate the efficacy and safety of TBC as a conditioning for ASCT in patients with CNS lymphoma. In this study, we aimed to assess the clinical outcomes of TBC/ASCT in both prospective and retrospective cohorts. Additionally, we compared the outcomes based on conditioning dose and disease status, analyzing prognostic factors in this setting.

## Materials and methods

### Participants and study design

An investigator-initiated, open-label, single-arm, prospective phase II trial was conducted at Seoul National University Hospital to evaluate the efficacy and safety of a high-dose TBC conditioning regimen followed by ASCT for CNS lymphoma (ClinicalTrials.gov identifier, NCT06625359). Patients older than 18 years with histologically and/or cytologically confirmed PCNSL or SCNSL were eligible. An Eastern Cooperative Oncology Group (ECOG) performance status (PS) of 0–2 and adequate organ function were required for eligibility. Patients who had previously received high-dose chemotherapy and ASCT were excluded.

The conditioning dose was adjusted based on age at ASCT and PS. For patients with a PS of 0–1 and age < 60 years, TBC conditioning was initiated on day − 9. Beginning on day − 9 through day − 7, each patient was treated with thiotepa (200 mg/m^2^ intravenously [IV] per day). On days − 6 to -4, patients received busulfan (2.7 mg/kg IV over 3 h per day). On days − 3 and − 2, cyclophosphamide (60 mg/kg IV per day) was given. Patients with a PS of 2 or age ≥ 60 years received busulfan (3.2 mg/kg IV over 3 h per day for 2 days), resulting in 8-day regimen.

This trial was terminated early due to slow recruitment after enrolling 17 patients between July 2015 and July 2021. A retrospective chart review of patients with CNS lymphoma treated at Seoul National University Hospital between January 2014 and July 2021 identified 18 patients who were not enrolled in this trial but received the same TBC conditioning and ASCT protocol. Following termination of the trial, our center standardized the TBC conditioning to an 8-day regimen for patients with CNS lymphoma, irrespective of age and PS. Nine patients were treated with the 8-day TBC conditioning regimen and ASCT between August 2021 and December 2023. In total, 44 patients were included in the study and classified into 8-day and 9-day groups according to the TBC regimen received (Figure S1).

### Transplantation procedures

Busulfan-related seizure prophylaxis was given with levetiracetam (1,500 mg loading on day − 6, 500 mg twice daily on days − 5 to -3). G-CSF was given daily at a dose of 300 mcg following the transplantation until the absolute neutrophil count (ANC) ≥ 1,000 for 3 consecutive days. The serum cytomegalovirus (CMV) polymerase chain reaction test was performed twice a week, and preemptive ganciclovir therapy was initiated in cases of CMV reactivation. The antiemetics, blood component, and other supportive care measures was used if applicable. Patients were discharged when there was no evidence of infection and they were clinically stable.

All patients received a uniform prophylactic regimen consisting of ciprofloxacin, trimethoprim/sulfamethoxazole, micafungin, and acyclovir. A thorough clinical and microbiological assessment was performed whenever patients showed any signs or symptoms of infection during hospitalization. Blood cultures and chest radiographs were conducted, and additional specimens from suspected infection sites were collected when clinically indicated [18].

### Definitions and outcomes

PCNSL was defined as lymphoma confined to the CNS, whereas SCNSL was defined as lymphoma with systemic disease and CNS involvement, or as initial diagnosis of systemic lymphoma followed by relapse in the CNS, regardless of systemic involvement at the time of relapse [19]. Immunohistochemical staining was prospectively performed on representative whole formalin-fixed, paraffin-embedded tissue sections for routine clinical diagnosis. The periventricular regions, basal ganglia, brain stem, corpus callosum, and cerebellum were considered deep structure regions of the brain. Neutrophil engraftment was defined as an ANC > 500 for 3 consecutive measurements. Platelet engraftment was defined as 3 consecutive measurements of > 20 × 10^9^/L without transfusion. Response was evaluated as complete response (CR), partial response (PR), stable disease (SD), or progressive disease (PD) according to the criteria of the International PCNSL Collaborative Group using magnetic resonance imaging (MRI) [20]. Non-relapse mortality (NRM) was defined as death without relapse or disease progression. Progression-free survival (PFS) and overall survival (OS) were calculated from the date of transplantation. Adverse events were assessed according to the National Cancer Institute Common Terminology Criteria for AE v5.0. Patients who need anticonvulsant during or after ASCT are considered to suffer from neurotoxicity.

### Statistical analysis

Descriptive statistics are presented as median values with ranges or numbers with percentiles. Differences between groups were assessed using Student’s t-test or Wilcoxon rank-sum test for continuous variables, and chi-square test for categorical variables, as indicated. Median follow-up duration was calculated by the reverse Kaplan-Meier method. PFS and OS were estimated by the Kaplan–Meier method and log-rank test was used for univariate comparisons of survival. Cumulative incidence curves were used in competing risk setting to calculate the probability of relapse and NRM. Univariate and multivariate Cox proportional hazards regression models were used to analyze OS. Multivariate analysis was performed using stepwise backward selection, and adjusted hazard ratios (HRs) with 95% confidence intervals (CIs) were calculated. All tests were 2-sided, and *P*-values of < 0.05 were considered statistically significant. The statistical software “R” version 4.3.1 (www.r-project.org) was used for all statistical analyses.

## Results

### Patient and disease characteristics

The study included 44 patients (median age, 57 years [range, 38–69]; 26 [59.1%] were male). Patients received either an 8-day regimen (*n* = 25), or a 9-day regimen (*n* = 19) for conditioning (Table 1). In total, 25 patients (56.8%) had PCNSL and 40 (90.9%) had diffuse large B-cell lymphoma (DLBCL) (Table S1). All patients were immunocompetent. As expected, patients in the 8-day group were older and had worse PS than those in the 9-day group; however, apart from age and PS, there were no significant differences in baseline characteristics between the two groups. There were no significant differences in disease type, age, or PS between the prospective and retrospective cohorts (Table S2).

**Table 1:** Baseline characteristics

	All patients *N* = 44	8-day *N* = 25	9-day *N* = 19	*P*-value
**Prospectively enrolled patient**,** n (%)**	17 (38.6)	5 (20.0)	12 (63.2)	0.009
**Disease**,** n (%)**				0.856
PCNSL	25 (56.8)	15 (60.0)	10 (52.6)	
SCNSL	19 (43.2)	10 (40.0)	9 (47.4)	
**Age at ASCT**,** median (range)**	57 (38–69)	61 (45–69)	49 (38–59)	< 0.001
Age ≥ 60 years, n (%)	14 (31.8)	14 (56.0)	0	< 0.001
**Sex**,** n (%)**				0.159
Male	26 (59.1)	12 (48.0)	14 (73.7)	
Female	18 (40.9)	13 (52.0)	5 (26.3)	
**Deep brain involvement**,** n (%)**	26 (59.1)	14 (56.0)	12 (63.2)	0.866
**Leptomeningeal involvement**,** n (%)**	12 (27.3)	7 (28.0)	5 (26.3)	1.000
**Histology**,** n (%)**				0.122
Burkitt lymphoma	2 (4.5)	0	2 (10.5)	
Diffuse large B-cell lymphoma	40 (90.9)	25 (100)	15 (78.9)	
Immunoblastic large B-cell lymphoma	1 (2.3)	0	1 (5.3)	
Not assessed	1 (2.3)	0	1 (5.3)	
**Immunohistochemistry profile**				
Bcl-2 (+), n (%)	23 (53.5)	14 (56.0)	9 (50.0)	0.937
Bcl-6 (+), n (%)	30 (69.8)	18 (72.0)	12 (66.7)	0.969
**Number of previous regimens**,** n (%)**				0.796
1	28 (63.6)	15 (60.0)	13 (68.4)	
≥ 2	16 (36.4)	10 (40.0)	6 (31.6)	
**Disease status at ASCT**,** n (%)**				0.615
CR1	19 (43.2)	10 (40.0)	9 (47.4)	
CR > 1	5 (11.4)	2 (8.0)	3 (15.8)	
PR	12 (27.3)	7 (28.0)	5 (26.3)	
SD/PD	8 (18.2)	6 (24.0)	2 (10.5)	
**ECOG PS at ASCT**,** n (%)**				0.036
0–1	37 (84.1)	18 (72.0)	19 (100)	
2	7 (15.9)	7 (28.0)	0	
**CD34 + cell**,** x10**^**6**^**/kg**,** median (range)**	4.98 (2.03–11.5)	4.47 (2.03–11.5)	5.52 (2.5-10.24)	0.090
**Time from diagnosis to ASCT**,** month**,** median (range)**	7.4 (1.3–96.1)	7.4 (1.3–96.1)	7.3 (2.6–48.2)	0.906

PCNSL, primary central nervous system lymphoma; SCNSL, secondary central nervous system lymphoma; ASCT, autologous stem cell transplantation; CR, complete remission; PR, partial remission; SD, stable disease; PD, progressive disease; ECOG PS, Eastern Cooperative Oncology Group performance status

All 25 patients with PCNSL had previously undergone first-line induction therapy with a methotrexate-based regimen, with 24 out of 25 patients receiving the methotrexate, vincristine, and procarbazine (MVP) and one patient receiving the methotrexate and cytarabine. Rituximab was added in 21 of 25 patients (84.0%). Three patients (12.0%) had undergone surgical resection of CNS mass. Of the 19 patients with SCNSL, 3 who had leptomeningeal involvement at the time of initial diagnosis were treated with rituximab, cyclophosphamide, doxorubicin, vincristine, and prednisolone (R-CHOP) along with intrathecal methotrexate. Of the remaining 16 patients with SCNSL who presented with CNS relapse, 15 were diagnosed based on isolated CNS involvement, and 11 received methotrexate-based chemotherapy as the initial CNS-directed treatment. In total, 13 patients (29.5%) patients had received WBRT before ASCT. Overall, 25 patients (56.8%) had undergone ASCT in CR (19 in CR1 and 6 in CR > 1), 13 (29.5%) in PR, and 6 (13.6%) in SD or PD.

### Transplantation outcomes and toxicities

Neutrophil engraftment (100% vs. 94.7%) and platelet engraftment (100% vs. 89.5%) did not differ between the two groups (Table 2). Overall, CMV antigenemia was observed in 5 patients, but no cases of CMV disease were identified. The median length of hospital stay was 15 days, with 4 patients staying longer than 30 days. Septic shock occurred in 7 patients (15.9%). Neurotoxicity occurred in a total of 5 patients; notably, all of them had received WBRT prior to ASCT.

**Table 2:** Transplantation outcome

	8-day *N* = 25	9-day *N* = 19	*P*-value
**Neutrophil engraftment**,** n (%)**	25 (100)	18 (94.7)	0.889
Time to engraftment, day, median (range)	11 (9–12)	11 (9–19)	0.788
**Platelet engraftment**,** n (%)**	25 (100)	17 (89.5)	0.352
Time to engraftment, day, median (range)	12 (9–24)	13 (7–23)	0.699
**Length of hospital stay**,** day**,** median (range)**	14 (9-112)	18 (6–27)	0.552
**CMV antigenemia**,** n (%)**	4 (16.0)	1 (5.3)	0.527
Time to CMV antigenemia, day, median (range)	32 (6–49)	16	
**CMV disease**,** n (%)**	0	0	
**Other complications**,** n (%)**			
Septic shock	3 (12.0)	4 (21.1)	0.691
Hemorrhagic cystitis	1 (4.0)	1 (5.3)	1.000
Veno-occlusive disease	0	0	
Transient neurotoxicity	1 (4.0)	2 (10.5)	0.805
Persistent neurotoxicity	2 (8.0)	0	0.595
**Remission status after ASCT**,** n (%)**			0.250
CR	18 (72.0)	14 (73.7)	
PR	1 (4.0)	0	
SD	1 (4.0)	0	
PD	4 (16.0)	1 (5.3)	
Not applicable	1 (4.0)	4 (21.1)	
**Relapse or progression**,** n (%)**	13 (52.0)	5 (26.3)	0.159
**Relapse at 3 years**,** % (95% CI)**	51.3 (25.5–68.1)	33.3 (4.7–53.4)	0.084
**PFS at 3 years**,** % (95% CI)**	46.5 (30.2–71.8)	52.6 (34.4–80.6)	0.490
**Death**,** n (%)**	9 (36.0)	6 (31.6)	1.000
**Cause of death**,** n (%)**	*N* = 9	*N* = 6	0.094
Disease	8 (88.9)	2 (33.3)	
Transplantation-related	1 (11.1)	4 (66.7)	
**OS at 3 years**,** % (95% CI)**	60.5 (43.0-85.1)	73.7 (56.3–96.4)	0.770
**Non-relapse mortality at 1 year**,** % (95% CI)**	4.6 (0-12.9)	21.1 (0.4–37.4)	0.073

CMV, cytomegalovirus; ASCT, autologous stem cell transplantation; CR, complete remission; PR, partial remission; SD, stable disease; PD, progressive disease; CI, confidence interval; PFS, progression-free survival; OS, overall survival

All 44 patients experienced febrile neutropenia in nadir; among them, infectious etiology was identified in 15 patients (Table 3). Overall, 28 infectious episodes were observed in these 15 patients, including 8 cases of bloodstream infection, 4 catheter-related infections, and 5 lung infections (Table S3). 46.4% were of bacterial, 42.9% were of viral, and 10.7% were of fungal origin. The majority of infections (60.0%) occurred during the pre-engraftment period (day 0–30), followed by the conditioning phase. Infections during the post-engraftment period (beyond day 30) were relatively uncommon. Excluding febrile neutropenia, 39 patients (88.6%) experienced at least 1 toxicity. The most common toxicities were diarrhea (61.4%), nausea/vomiting (47.7%), and oral mucositis (36.4%).

**Table 3:** Toxicities

	All patients *N* = 44	8-day *N* = 25	9-day *N* = 19
**Infections**	15 (34.1)	10 (40.0)	5 (26.3)
**Bacterial infection**,** n (%)**	10 (22.7)	6 (24.0)	4 (21.1)
Grade 3 or 4	7 (15.9)	3 (12.0)	4 (21.1)
**Viral infection**,** n (%)**	9 (20.5)	6 (24.0)	3 (15.8)
Grade 3 or 4	7 (15.9)	4 (16.0)	3 (15.8)
**Fungal infection**,** n (%)**	3 (6.8)	3 (12.0)	0
Grade 3 or 4	3 (6.8)	3 (12.0)	0
**Nausea/vomiting**,** n (%)**	21 (47.7)	9 (36.0)	12 (63.2)
Grade 3 or 4	7 (15.9)	3 (12.0)	4 (21.1)
**Diarrhea**,** n (%)**	27 (61.4)	13 (52.0)	14 (73.7)
Grade 3 or 4	5 (11.4)	2 (8.0)	3 (15.8)
**Constipation**,** n (%)**	2 (4.5)	1 (4.0)	1 (5.3)
Grade 3 or 4	0	0	0
**Oral Mucositis**,** n (%)**	16 (36.4)	8 (32.0)	8 (42.1)
Grade 3 or 4	3 (6.8)	3 (12.0)	0
**Skin rash**,** n (%)**	5 (11.4)	3 (12.0)	2 (10.5)
Garde 3 or 4	0	0	0
**Dyspnea**,** n (%)**	4 (9.1)	2 (8.0)	2 (10.5)
Garde 3 or 4	3 (6.8)	2 (8.0)	1 (5.3)
**Neuropathy**,** n (%)**	4 (9.1)	1 (4.0)	3 (15.8)
Garde 3 or 4	0	0	0
**Seizure**,** n (%)** Garde 3 or 4	1 (2.3) 0	1 (4.0) 0	0 0
**Delirium**,** n (%)**	9 (20.5)	7 (28.0)	2 (10.5)
Garde 3 or 4	4 (9.1)	3 (12.0)	1 (5.3)
**Creatinine elevation**,** n (%)**	7 (15.9)	3 (12.0)	4 (21.1)
Garde 3 or 4	4 (9.1)	2 (8.0)	2 (10.5)
**Bilirubin elevation**,** n (%)**	2 (4.5)	0	2 (10.5)
Garde 3 or 4	2 (4.5)	0	2 (10.5)
**AST/ALT elevation**,** n (%)**	4 (9.1)	3 (12.0)	1 (5.3)
Garde 3 or 4	1 (2.3)	1 (4.0)	0

AST, aspartate aminotransferase; ALT, alanine aminotransferase

Up to September 2024, all patients were followed for a median of 59.0 months (95% CI, 37.9–65.0). Following ASCT, 33 of 44 patients (75.0%) achieved an objective response (32 CR and 1 PR). During follow-up, 18 patients (40.9%) relapsed and 15 (34.1%) died. We observed five (11.4%) treatment-related deaths after ASCT, all of which were attributed to septic shock (Table S4). Among these, four patients in the 9-day group died within one month, while remaining patient in the 8-day group died 1.6 months post-ASCT due to *Pneumocystis* pneumonia.

### Survival outcomes

**Fig. 1:**
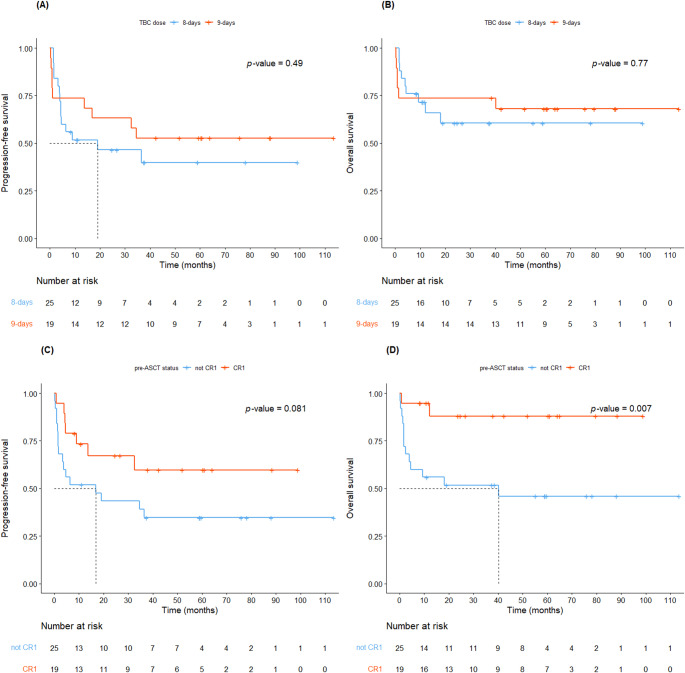
Survival outcomes based on conditioning dose and disease status. (**A**) Progression-free survival (PFS) by conditioning dose; (**B**) Overall survival (OS) by conditioning dose; (**C**) PFS by pre-transplantation disease status; (**D**) OS by pre-transplantation disease status

For the entire cohort, median PFS was 34.6 months, while median OS was not reached (Figure S2). The 3-year PFS and OS probabilities were 48.0% (95% CI, 34.9–66.0) and 67.2% (95% CI, 54.4–83.0), respectively. The 3-year probabilities of PFS (46.5% vs. 52.6%, *P* = 0.49) and OS (60.5% vs. 73.7%, *P* = 0.77) for 8-day and 9-day groups were comparable (Fig. 1A and B). The 3-year PFS for patients in CR1 was numerically higher than that for patients not in CR1, although the difference was not statistically significant (Figs. 1C and 59.7% vs. 39.0%, *P* = 0.081). The 3-year OS for patients in CR1 was significantly higher than that for patients not in CR1 (Figs. 1D and 88.0% vs. 51.7%, *P* = 0.007). There were no statistically significant differences in PFS and OS according to disease type (Table S5, Figure S3).

On univariate analysis, Bcl-6 (HR 0.28; 95% CI, 0.10–0.79; *P* = 0.005), disease status (HR 0.18; 95% CI, 0.04–0.78; *P* = 0.022) and PS (HR 3.20; 95% CI, 1.07–9.50; *P* = 0.037) were statistically significant factors for OS (Table 4). The multivariate Cox proportional-hazards model showed that Bcl-6 positivity (HR 0.26; 95% CI, 0.09–0.74; *P* = 0.011) and CR1 at ASCT (HR 0.16, 95% CI 0.04–0.73, *P* = 0.018) were favorable indicators of OS.

**Table 4:** Cox proportional hazard model for overall survival

	Univariable		Multivariable	
	HR (95% CI)	*P*-value	HR (95% CI)	*P*-value
**Age at ASCT ≥ 60**	0.76 (0.24–2.42)	0.648		
**Group**				
8-day regimen	Ref			
9-day regimen	0.92 (0.32–2.62)	0.869		
**Disease**				
PCNSL	Ref			
SCNSL	1.08 (0.39–2.99)	0.877		
**Deep brain involvement**	0.80 (0.29–2.20)	0.658		
**Leptomeningeal involvement**	2.25 (0.80–6.37)	0.125		
**Bcl-2 (+)**	1.86 (0.63–5.46)	0.258		
**Bcl-6 (+)**	0.28 (0.10–0.79)	0.016	0.26 (0.09–0.74)	0.011
**Disease status at ASCT**				
Not in CR1	Ref		Ref	
In CR1	0.18 (0.04–0.78)	0.022	0.16 (0.04–0.73)	0.018
**ECOG PS at ASCT**				
2	Ref			
0–1	0.31 (0.11–0.93)	0.037		
**CD34 + cell > 4.98 × 10** ^**6**^ **/kg**	0.82 (0.30–2.25)	0.694		

HR, hazard ratio; CI, confidence interval; ASCT, autologous stem cell transplantation; PCNSL, primary central nervous system lymphoma; SCNSL, secondary central nervous system lymphoma; CR, complete remission; ECOG PS, Eastern Cooperative Oncology Group performance status

**Fig. 2:**
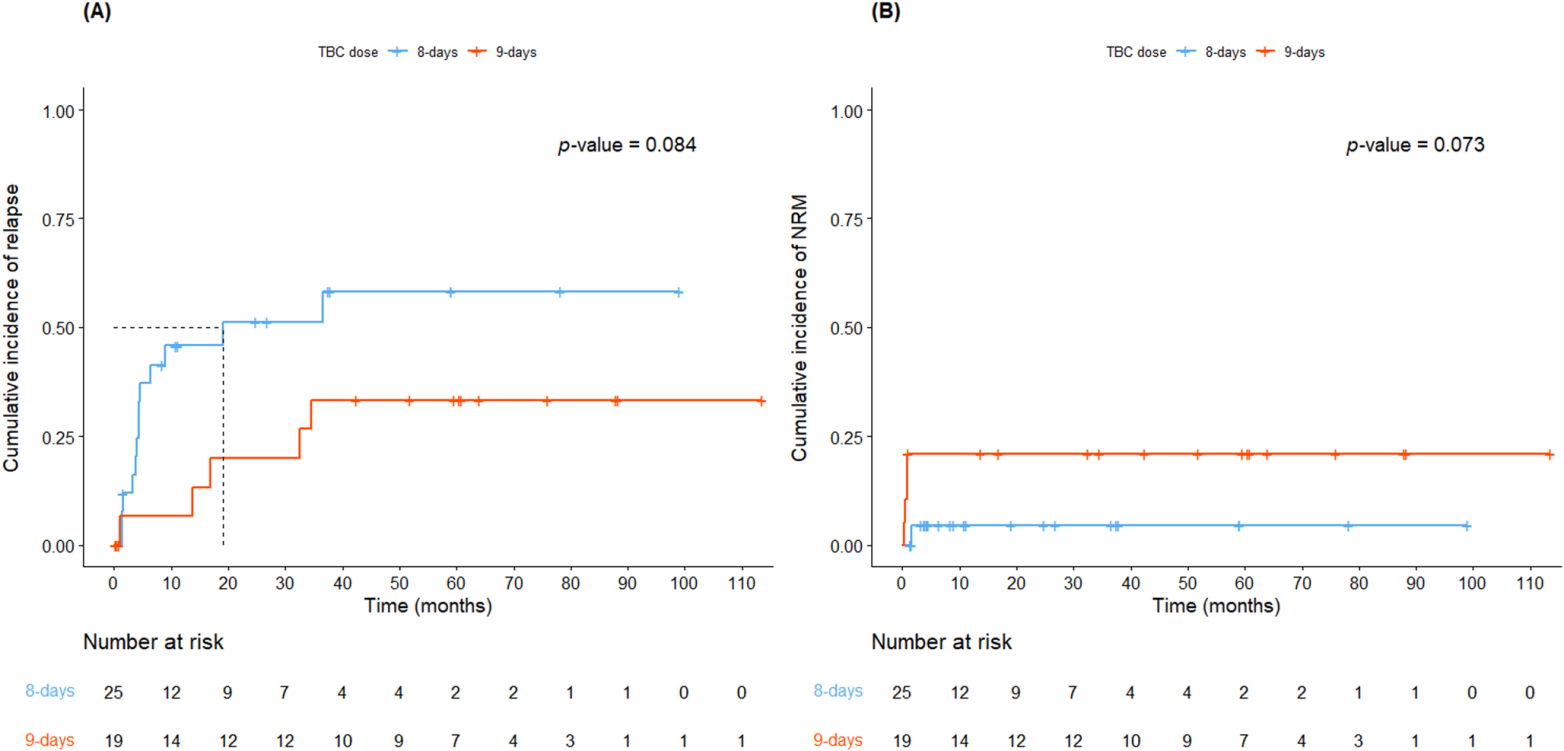
Cumulative incidence of relapse and non-relapse mortality (NRM). (**A**) Relapse by conditioning dose; (**B**) NRM by conditioning dose

The 3-year cumulative incidence of relapse in the entire cohort was 45.7% (95% CI, 26.7–59.8). The 3-year cumulative incidence of relapse in the 8-day group showed a trend toward increase compared to that in the 9-day group (Figs. 2A and 51.3% vs. 33.3%, *P* = 0.084). The 1-year cumulative incidence of NRM in the entire cohort was 11.6% (95% CI, 1.5–20.7). The 1-year cumulative incidence of NRM in the 8-day group showed a trend toward decrease compared to that in the 9-day group (Figs. 2B and 4.6% vs. 21.1%, *P* = 0.073). There were no statistically significant differences in cumulative incidence of relapse and NRM according to disease type (Table S5, Figure S4).

## Discussion

In this study, we investigated the efficacy and safety of TBC/ASCT for patients with CNS lymphoma. In 44 patients who underwent ASCT following a uniform TBC conditioning as first-line consolidation or salvage therapy after relapse, the 3-year PFS and OS rates were 48.0% and 67.2%, respectively. Furthermore, we customized the intensity of TBC based on age and PS. We found that there was no difference in PFS and OS according to TBC intensity; however, despite the 9-day regimen group being younger and fitter, there was a tendency towards higher TRM in this group. The results of the present study suggest that dose modification of the standard TBC conditioning regimen may be needed, particularly for Asian populations.

Given the neurotoxicity risk associated with WBRT, high-dose chemotherapy followed by ASCT has increasingly become the preferred consolidation therapy for CNS lymphoma [15, 21]. Although hematopoietic stem cell transplantation is a curative treatment for hematologic malignancies [22, 23], no established conditioning regimen currently exists for CNS lymphoma. Based on its efficacy in refractory DLBCL, the BEAM (carmustine, etoposide, cytarabine, and melphalan) conditioning was adopted for PCNSL but demonstrated suboptimal outcomes. In a phase II trial involving 14 patients with PCNSL who underwent BEAM/ASCT, more than half of the patients experienced early relapse [24]. The limited CNS penetration of BEAM regimen may have contributed to this poor outcome; in contrast, thiotepa is known to penetrate the CNS well [25]. Following reports of the efficacy of TBC/ASCT in primary or secondary CNS lymphoma [26, 27], recent studies have explored several thiotepa-based conditioning regimens in CNS lymphoma (Table 5). Long-term data from a randomized phase II trial evaluating TBC/ASCT in PCNSL (PRECIS study) has recently shown the efficacy of TBC conditioning [28]. Unlike prospective studies involving highly selected patients, real-world data on TBC/ASCT in CNS lymphoma have reported various outcomes. Studies conducted in patients with better disease control than in our study have reported more favorable survival outcomes [29–33]. Notably, for patients with PCNSL who underwent ASCT in CR1, the 2-year OS was 95%, with a TRM of 2.9%, demonstrating excellent efficacy [32]. TT-BCNU (thiotepa, and carmustine) has been investigated in several studies as another CNS-directed conditioning regimen. IELSG-32 study, a randomized phase II trial, compared the efficacy of consolidation with either TT-BCNU/ASCT or WBRT; the 2-year PFS and OS after ASCT were 69% and 71%, respectively [15, 34]. Other studies on TT-BCNU/ASCT have shown similar results, with TRM rates around 3% [35, 36]. These findings suggest that while the efficacy of TT-BCNU conditioning may be somewhat lower compared to TBC, it could offer advantages in terms of reduced toxicity. While there are no prospective studies comparing the efficacy of thiotepa-based conditioning regimens, several retrospective reports are available. A recent study comparing BEAM and three different thiotepa-based regimens (TBC, TT-BCNU, and TT-Bu [thiotepa, and busulfan]) demonstrated that thiotepa-based regimens were superior to BEAM [16]. Among the thiotepa-based regimens, TBC and TT-Bu tended to offer better disease control compared to TT-BCNU. Alnahhas et al. reported that while TT-BCNU was inferior to TBC in terms of survival outcomes, it had the lowest TRM [37]. Scordo et al. also demonstrated that TBC was associated with a higher risk of NRM compared to TT-BCNU, but in patients under 60 years, TBC provided better survival outcomes than TT-BCNU [17]. The higher NRM risk associated with TBC, along with poor CNS penetration of cyclophosphamide compared to thiotepa and busulfan [25, 38], has led to the development of TT-Bu regimen, which excludes cyclophosphamide from TBC. Sanders et al. reported the safety and efficacy of TT-Bu conditioning followed by ASCT in PCNSL; there was no case of NRM [39]. In contrast, a recent prospective study on rituximab and TT-Bu conditioning reported lower survival outcomes and a NRM of 13.9% compared to the previous study [40]. Considering the efficacy and toxicity of each regimen, a strategy for selecting conditioning regimens based on patient tolerability may be necessary. Prospective randomized trials comparing thiotepa-based conditioning regimens in this regard are warranted.

**Table 5:** Compendium of publications on thiotepa-based conditioning followed by ASCT in CNS lymphoma

Study	Conditioning Regimen	No. of ASCT/ Study design	Disease	Age at ASCT (range)	ASCT setting	Disease status at ASCT	Median follow-up	EFS/PFS	OS	TRM
Our study	TBC	*N* = 44, retrospective	PCNSL, SCNSL	57 (38–69)	1st line & R/R	CR 54.5%, PR 27.3%	59	3 year, 48.0%	3 year, 67.2%	11.6%
Delphine et al. (2023) [29]	TBC	*N* = 24, retrospective	PCNSL, SCNSL	58 (23–66)	1st line & R/R	CR 63%, PR 38%	10	2 year, 64%	2 year, 75%	21%
Scordo et al. (2021) [17]	TBC TT-BCNU	*N* = 263 *N* = 275, retrospective	PCNSL	59 (23–76) 60 (20–78)	1st line & R/R	CR1 54% CR1 60%	36 24	3 year, 75% 3 year, 76%	3 year, 81% 3 year, 78%	11% 4%
Young et al. (2020) [28]	TBC	*N* = 48, retrospective	PCNSL, SCNSL	59 (IQR, 52–66)	1st line & R/R	CR1 60%	23.9	2 year, 80.5%	2 year, 80.1%	8.3%
Scordo et al. (2017) [30]	TBC	*N* = 43, retrospective	PCNSL, SCNSL	56 (25–71)	1st line & R/R	CR 81%, PR 19%	20	1 year, 83%	1 year, 87%	7%
DeFilipp et al. (2017) [31]	TBC	*N* = 46, retrospective	PCNSL	59 (27–69)	1st line	CR1 100%	32	2 year, 92%	2 year, 95%	2.9%
Houillier et al. (2022) [27]	TBC	*N* = 44, prospective	PCNSL	55 (25–60)	1st line	CR1 50%, PR 34%	98	8 year, 67%	8 year, 69%	11.4%
Qualls et al. (2017) [32]	TBC	*N* = 20, retrospective	SCNSL	54 (38–67)	1st line	CR1 100%	52.8	4 year, 77%	4 year, 82%	5%
Schorb et al. (2017) [34]	Mostly TT-BCNU	*N* = 52, retrospective	PCNSL	68 (65–77)	1st line & R/R	CR 34.6%, PR 51.9%	22.1	2 year, 62%	2 year, 70.8%	3.8%
Ferreri et al. (2017) [15]	TT-BCNU	*N* = 59, prospective	PCNSL	58 (26–70)	1st line	CR1 53%, PR/SD 47%	40	2 year, 69%	2 year, 71%	3.4%
Khurana et al. (2020) [35]	TT-BCNU	*N* = 31, retrospective	PCNSL	61 (IQR, 57–65)	1st line & R/R	CR 71%	24	2 year, 65.5%	2 year, 81.6%	3.2%
Schorb et al. (2024) [39]	R-TT-Bu	*N* = 36, prospective	PCNSL	71 (65–80)	1st line	CR/PR/SD 100%	23	2 year, 73.7	2 year, 80.6	13.9%
Sanders et al. (2019) [38]	TT-Bu	*N* = 30, retrospective	PCNSL	58 (39–68)	1st line	CR1 34%, PR 53%	19.7	3 year, 88.9%	3 year, 92.7%	0%

ASCT, autologous stem cell transplantation; EFS, event-free survival; PFS, progression-free survival; OS, overall survival; TRM, transplant-related mortality; PCNSL, primary central nervous system lymphoma; SCNSL, secondary nervous system lymphoma; IQR, interquartile range; R/R, relapsed and/or refractory; CR, complete remission; PR, partial remission; SD, stable disease; TBC, thiotepa/busulfan/cyclophosphamide; TT-BCNU, thiotepa/carmustine; R-TT-BU, rituximab/thiotepa/busulfan

There is a concern that TBC conditioning may be related to a higher risk of toxicities and infections compared to other thiotepa-based conditioning regimens [41, 42]. In particular, the NRM risk is high in patients over 60 years of age [17]. Therefore, there have been attempts to adjust the busulfan dose in TBC conditioning based on the age of 60. DeFilipp et al. conducted TBC/ASCT in patients with PCNSL, using a busulfan total dose of 8.0 to 9.6 mg/kg for those under 60 years of age and 7.2 mg/kg for those over 60, which is slightly higher than the dose used in our study [32]. They reported excellent outcomes, with a 2-year survival rate exceeding 90% and a NRM of 2.9%; however, all ASCT were performed in CR1, which likely contributed to the favorable outcomes. In contrast, Delphine et al. reported a 2-year OS of 78% with NRM of 21% in TBC/ASCT using a total busulfan dose of 8.0 mg/kg for patients under 60 years old, which is similar to the dose we used [30]. In our study conducted with a similar population, documented infections occurred in 15 patients, and septic shock occurred in 7 patients. The profile of toxicities and infections is consistent with previous studies [30–32]. Overall, 5 patients died from treatment-related death, resulting in a NRM of 11.6%. Despite the 9-day regimen group being younger and fitter, there was a tendency toward a higher NRM of 21.1% compared to 4.6% in the 8-day regimen group. All 4 cases of NRM in the 9-day group were due to septic shock, with deaths occurring within one month of ASCT. In addition, glutathione S-transferase polymorphisms are relatively common in Asian populations, which may lead to increased busulfan exposure [43, 44]. Taken together, dose modification of the conventional busulfan dose in the TBC regimen may be necessary, particularly in Asian populations, and further research on optimal dosing is warranted.

Prognostic factors have been an area of research due to the poor prognosis of CNS lymphoma. We found that ASCT at CR1 is a favorable prognostic factor. European and American studies on PCNSL have also reported that performing ASCT as part of first-line treatment in CR is a favorable prognostic factor [16, 29, 36]. ASCT as consolidation therapy is recommended for patients in CR, and the role of ASCT in CR1 requires further investigation. In contrast, age and PS were not associated with survival outcome, which aligns with previous studies [16, 45]. These findings suggest that high-dose chemotherapy followed by ASCT is an effective treatment not only for fit patients under 60 but also for those over 60 with CNS lymphoma. Further exploration of the optimal regimen and dosing in this population is warranted. In addition, our data showed Bcl-6 expression is associated with longer OS. While Bcl-6 expression is a favorable prognostic factor in non-CNS DLBCL [46], its prognostic impact in PCNSL has shown conflicting results in previous studies. Several previous studies found that Bcl-6 expression correlates with adverse outcomes [47, 48]. Bcl-6 may appear to be a poor prognostic indicator due to its association with other unfavorable factors, such as c-Myc expression and old age [49, 50]. The favorable impact of Bcl-6 on survival in our data may have been influenced by the inclusion of patients with SCNSL. However, there are recent studies that have reported Bcl-6 as a favorable prognostic factor in PCNSL [51]. The retrospective nature, different treatment protocols and varying immunophenotype methods may have contributed to these inconsistent results. Although DLBCL with CNS relapse frequently exhibit the MCD subtype, resembling the molecular features of PCNSL, the distinct disease entities and their respective biological contexts should also be taken into account [52]. Therefore, further studies regarding biomarkers in CNS lymphoma are warranted.

This study has several limitations. First, this was a single-center, retrospective study without a concurrent control arm. Although we initiated a prospective phase II trial, the number of enrolled patients was limited. Implementing TBC/ASCT on a consistent platform for CNS lymphoma remains challenging due to the rarity of the disease. Furthermore, although we performed various analyses, including multivariate Cox analysis, the inclusion of both prospective and retrospective cohorts may have introduced potential selection bias. Second, neuropsychological evaluations were not performed prospectively, which may have led to an underestimation of subtle cognitive impairment. Given the nature of this disease entity, neurocognitive outcomes are an important consideration in determining treatment strategies. Although some reports suggest that high-dose chemotherapy followed by ASCT does not results in neurocognitive impairment in these patients [53], further research is needed to evaluate long-term outcomes related to quality of life and functional status.

Despite these limitations, our study evaluates the efficacy and safety of ASCT in patients with primary or secondary CNS lymphoma, with consistent TBC conditioning. We initiated a prospective study comparing the conventional dose (9-day regimen) and the modified dose (8-day regimen) of TBC conditioning, and found that the modified regimen reduced NRM without compromising survival outcomes. In addition, we highlight the CR1 at ASCT as a favorable prognostic indicator, advocating for early ASCT in CNS lymphoma. For refractory CNS lymphoma, a condition with a poor prognosis, recent data on immunochemotherapies are lacking. Further research is imperative to develop improved treatment modalities for this challenging disease.

In conclusion, our study demonstrates the efficacy of TBC/ASCT in CNS lymphoma in the Asian population. Notably, the conventional busulfan dose may be associated with higher toxicity and TRM, highlighting the potential need for a modified TBC regimen in Asian populations. Although thiotepa-based conditioning appears superior to BEAM, no randomized controlled trials have compared the various thiotepa-based regimens, representing a clinical unmet need. Further studies are required to identify the optimal thiotepa-based conditioning regimen that balances efficacy and safety in CNS lymphoma.

## Electronic supplementary material

Below is the link to the electronic supplementary material.

Supplementary Material 1

## Data Availability

No datasets were generated or analysed during the current study.
