# Supplementary material for: Autologous stem cell transplantation with thiotepa, busulfan, and cyclophosphamide conditioning in patients with central nervous system lymphoma: a phase II study
Source: Ann Hematol. 2025 Jun 14;104(7):3843–54. doi: 10.1007/s00277-025-06405-y (PMC12334454; doi:10.1007/s00277-025-06405-y)
Supplement: Supplementary file 1 — Supplementary Material 1 [file 277_2025_6405_MOESM1_ESM.docx]

**Supplementary Appendix**

**Autologous stem cell transplantation with thiotepa, busulfan, and cyclophosphamide conditioning in patients with central nervous system lymphoma: a phase II study**

Dong Hyun Kim, Taekeun Park, Junshik Hong, Dong-Yeop Shin, Inho Kim, Sung-Soo Yoon, Ja Min Byun, and Youngil Koh

**Correspondence to:**

Ja min Byun, MD, PhD

Department of Internal Medicine, Seoul National University Hospital, 101, Daehak-ro, Jongro-gu, Seoul 03080, Republic of Korea

Tel +82-2-2072-7215

E-mail [jaminbyun@snu.ac.kr](mailto:jaminbyun@snu.ac.kr)

Youngil Koh, MD, PhD

Department of Internal Medicine, Seoul National University Hospital, 101, Daehak-ro, Jongro-gu, Seoul 03080, Republic of Korea

Tel +82-2-2072-7217

E-mail [go01@snu.ac.kr](mailto:go01@snu.ac.kr)

**Supplementary Figure**

**Figure S1.** Scheme of TBC/ASCT.

**
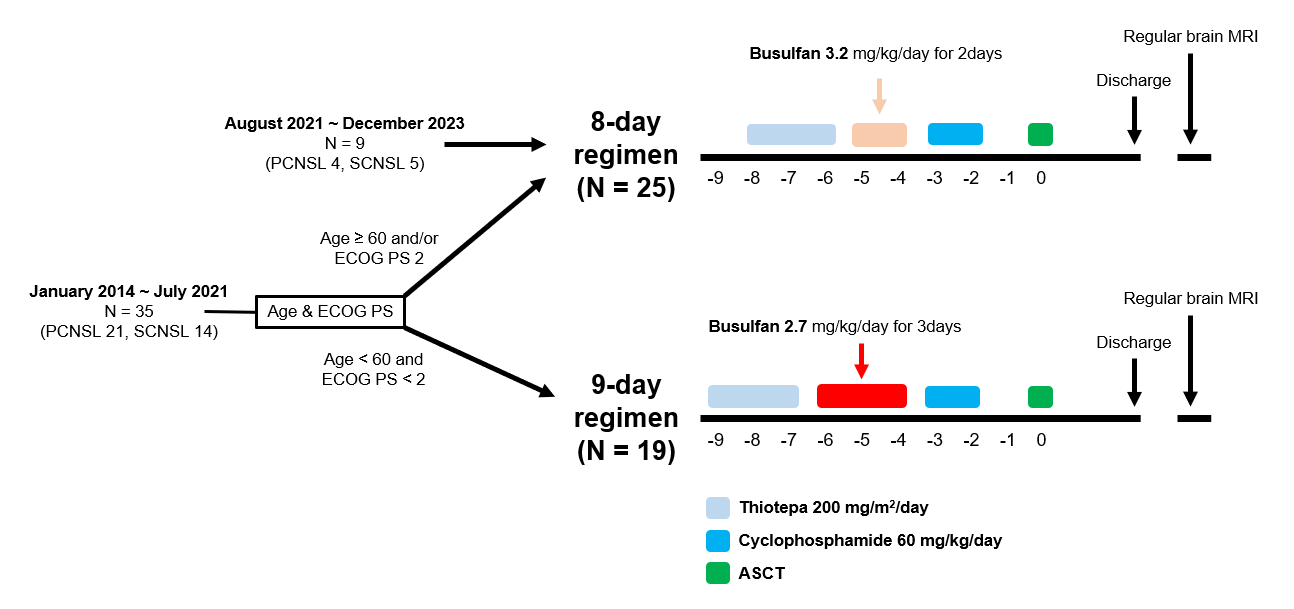
**

**Figure S2.** Survival outcomes in the entire patient cohort. (A) Progression-free survival; (B) Overall survival.


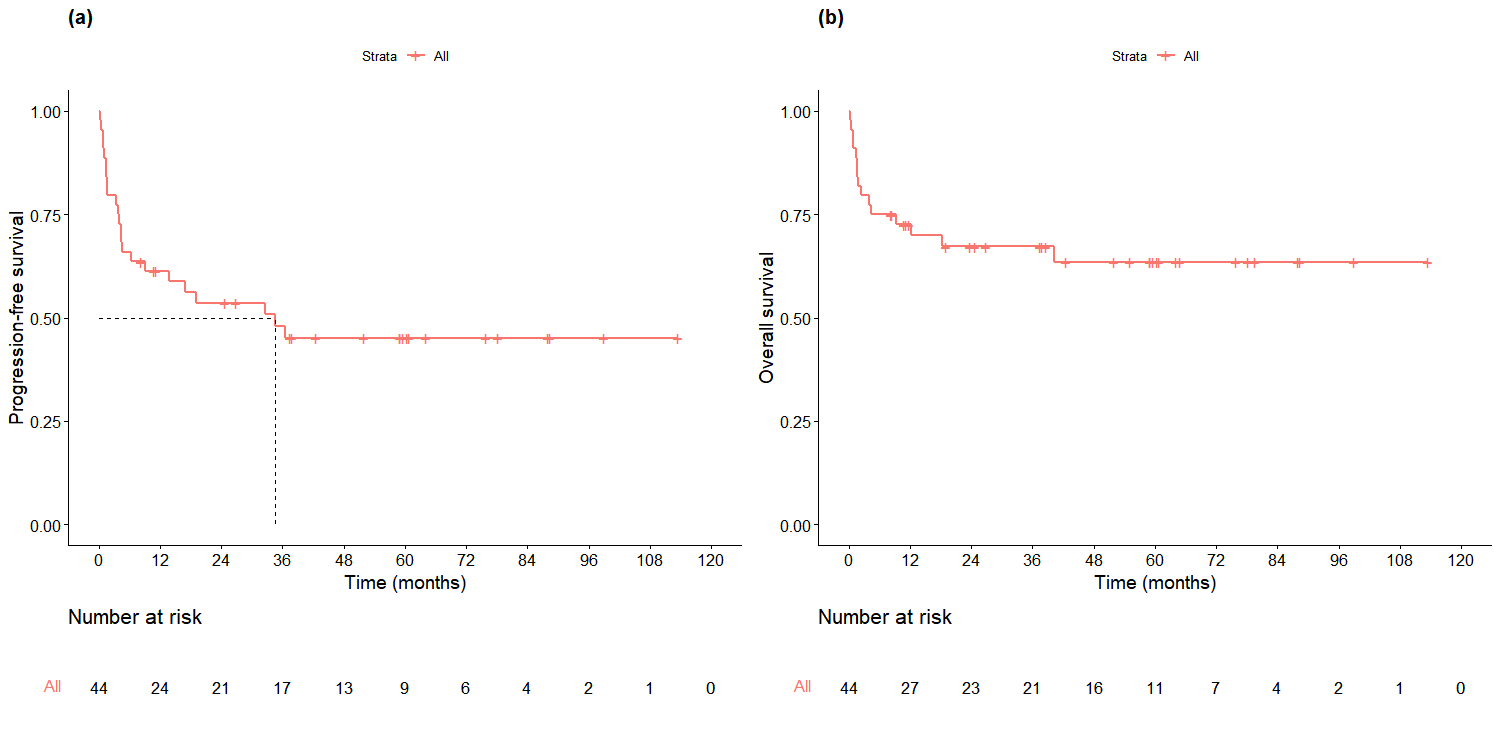


**Figure S3.** Survival outcomes according to disease type (PCNSL vs. SCNSL). (A) Progression-free survival; (B) Overall survival.


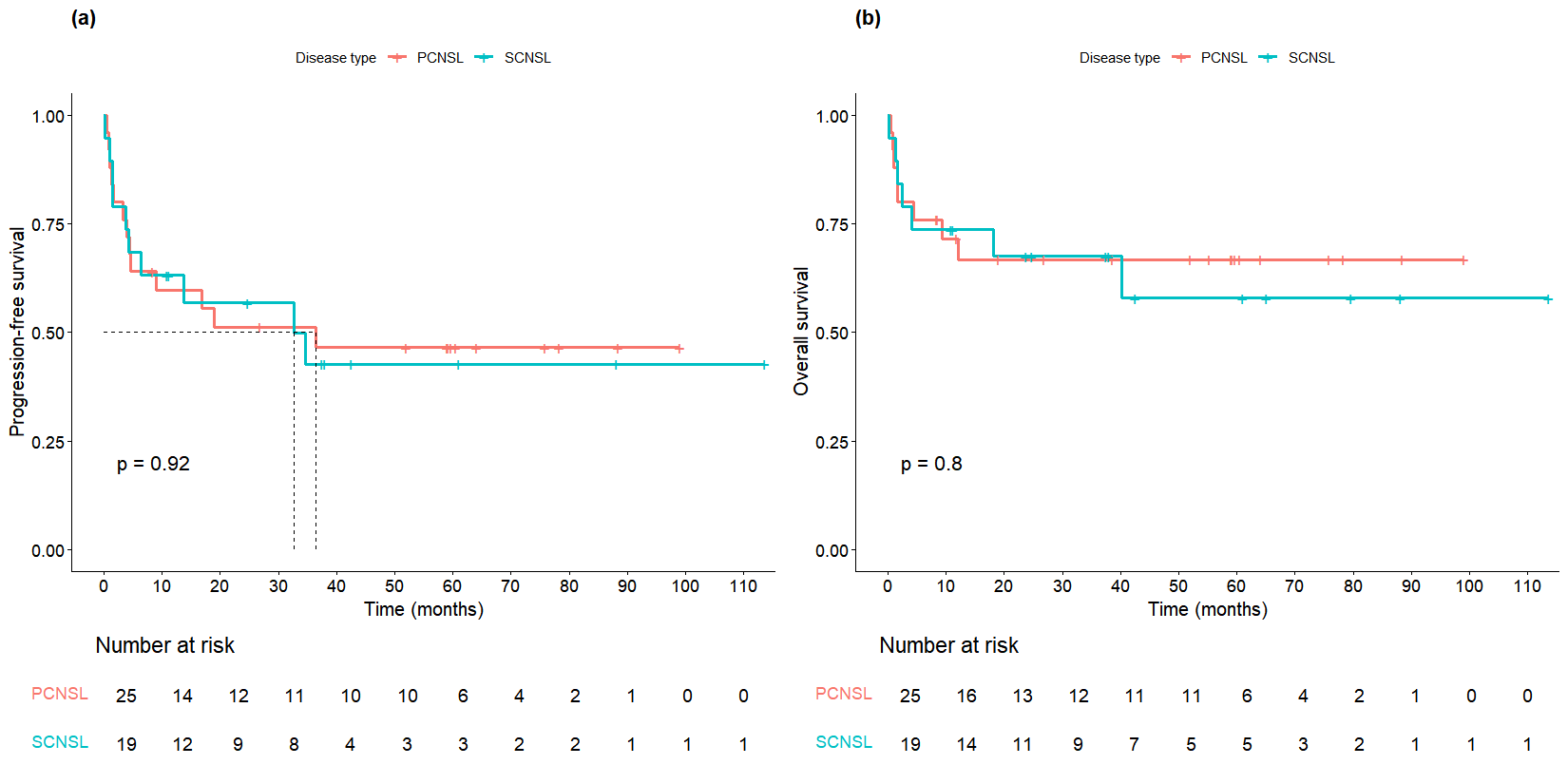


**Figure S4.** Cumulative incidence of relapse and non-relapse mortality (NRM) according to disease type (PCNSL vs. SCNSL). (A) Relapase; (B) NRM.


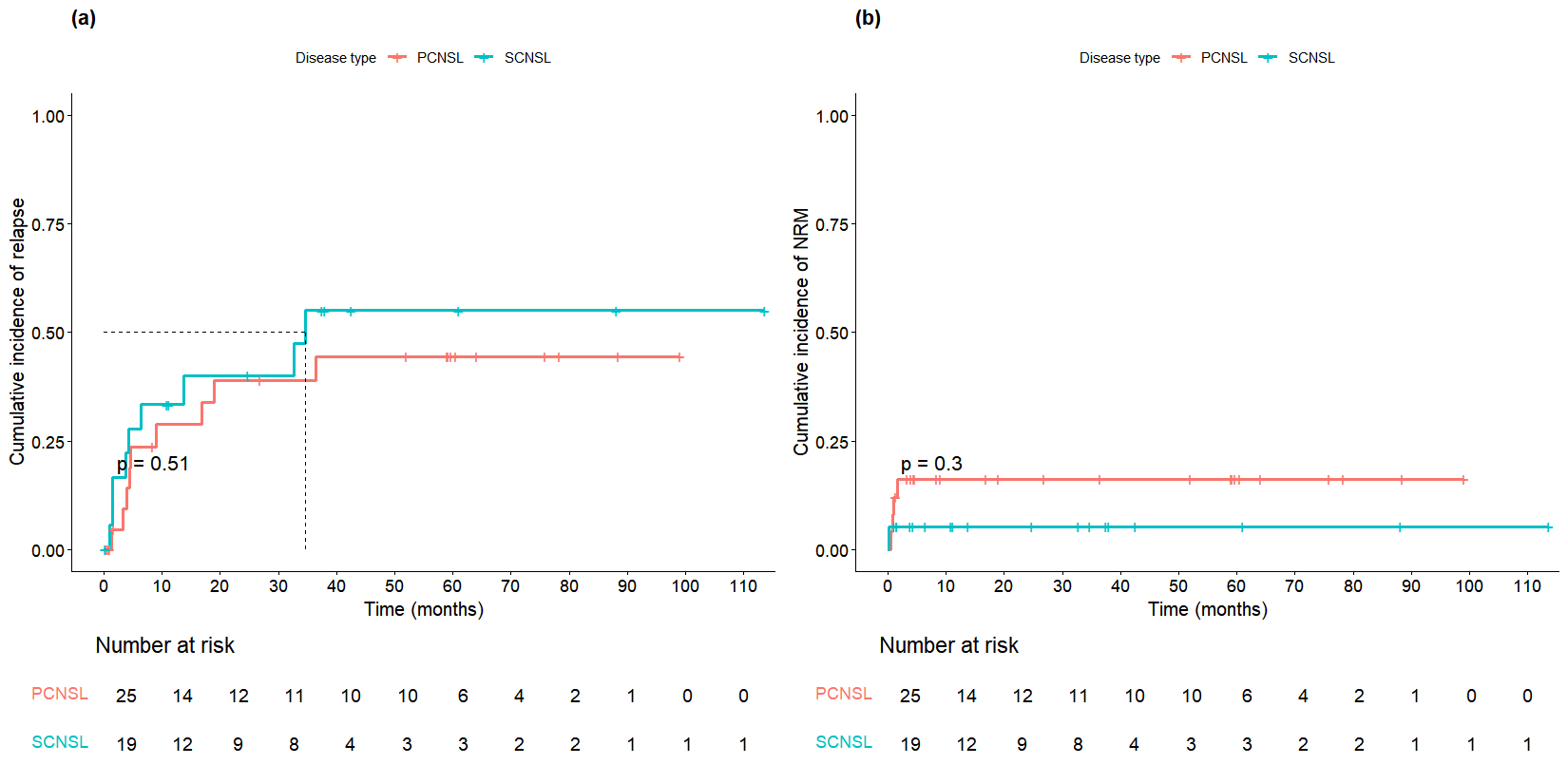


**Supplementary Table**

**Table S1.** Baseline characteristics according to disease type (PCNSL vs. SCNSL).

|  | **PCNSL**  **N = 25** | **SCNSL**  **N = 19** | ***P*-value** |
| --- | --- | --- | --- |
| **Prospectively enrolled patient, n (%)** | 12 (48.0) | 5 (26.3) | 0.250 |
| **Age at ASCT, median (range)**  Age ≥ 60 years, n (%) | 57 (40-69)  8 (32.0) | 57 (38-64)  6 (31.6) | 0.354  1.000 |
| **Sex, n (%)**  Male  Female | 17 (68.0)  8 (32.0) | 9 (47.4)  10 (52.6) | 0.285 |
| **Deep brain involvement, n (%)** | 17 (68.0) | 9 (47.4) | 0.285 |
| **Leptomeningeal involvement, n (%)** | 2 (8.0) | 10 (52.6) | 0.003 |
| **Histology, n (%)**  Burkitt lymphoma  Diffuse large B-cell lymphoma  Immunoblastic large B-cell lymphoma  Not assessed | 0  23 (92.0)  1 (4.0)  1 (4.0) | 2 (10.5)  17 (89.5)  0  0 | 0.245 |
| **Immunohistochemistry profile, n (%)**  Bcl-2 (+)  Bcl-6 (+) | 13 (54.2)  19 (79.2) | 10 (52.6)  11 (57.9) | 1.000  0.240 |
| **Number of previous regimens, n (%)**  1  ≥ 2 | 13 (52.0)  12 (48.0) | 15 (78.9)  4 (21.1) | 0.127 |
| **Disease status at ASCT, n (%)**  CR1  CR>1  PR  SD/PD | 11 (44.0)  4 (16.0)  7 (28.0)  3 (12.0) | 8 (42.1)  1 (5.3)  5 (26.3)  5 (26.3) | 0.506 |
| **ECOG PS at ASCT, n (%)**  0-1  2 | 21 (84.0)  4 (16.0) | 16 (84.2)  3 (15.8) | 1.000 |
| **CD34+ cell, x10^6^/kg, median (range)** | 3.50 (2.03-11.50) | 5.52 (2.35-10.24) | 0.044 |
| **Time from diagnosis to ASCT, month, median (range)** | 8.9 (4.7-96.1) | 5.8 (1.3-26.0) | 0.004 |

PCNSL, primary central nervous system lymphoma; SCNSL, secondary central nervous system lymphoma; ASCT, autologous stem cell transplantation; CR, complete remission; PR, partial remission; SD, stable disease; PD, progressive disease; ECOG PS, Eastern Cooperative Oncology Group performance status.

**Table S2.** Baseline characteristics according to disease type (PCNSL vs. SCNSL).

|  | **Prospective**  **N = 17** | **Retrospective**  **N = 27** | ***P*-value** |
| --- | --- | --- | --- |
| **Age at ASCT, median (range)**  Age ≥ 60 years, n (%) | 53 (38–64)  4 (23.5) | 57 (40–69)  10 (37.0) | 0.175  0.546 |
| **Sex, n (%)**  Male  Female | 11 (64.7)  6 (35.3) | 15 (55.6)  12 (44.4) | 0.775 |
| **Deep brain involvement, n (%)** | 13 (76.5) | 13 (48.1) | 0.122 |
| **Leptomeningeal involvement, n (%)** | 3 (17.6) | 9 (33.3) | 0.430 |
| **Disease type, n (%)**  PCNSL  SCNSL | 12 (70.6)  5 (29.4) | 13 (48.1)  14 (51.9) | 0.250 |
| **Histology, n (%)**  Burkitt lymphoma  Diffuse large B-cell lymphoma  Immunoblastic large B-cell lymphoma  Not assessed | 2 (11.8)  13 (76.5)  1 (5.9)  1 (5.9) | 0  27 (100)  0  0 | 0.072 |
| **Immunohistochemistry profile, n (%)**  Bcl-2 (+)  Bcl-6 (+) | 11 (68.8)  12 (75.0) | 12 (44.4)  18 (66.7) | 0.219  0.817 |
| **Number of previous regimens, n (%)**  1  ≥ 2 | 10 (58.8)  7 (41.2) | 18 (66.7)  9 (33.3) | 0.838 |
| **Disease status at ASCT, n (%)**  CR1  CR>1  PR  SD/PD | 6 (35.3)  4 (23.5)  7 (41.2)  0 | 13 (48.1)  1 (3.7)  5 (18.5)  8 (29.6) | 0.012 |
| **ECOG PS at ASCT, n (%)**  0-1  2 | 16 (94.1)  1 (5.9) | 21 (77.8)  6 (22.2) | 0.308 |
| **CD34+ cell, x10^6^/kg, median (range)** | 5.81 (2.35-11.50) | 4.82 (2.03-10.20) | 0.214 |
| **Time from diagnosis to ASCT, month, median (range)** | 7.9 (2.6-46.6) | 7.0 (1.3-96.1) | 0.656 |

PCNSL, primary central nervous system lymphoma; SCNSL, secondary central nervous system lymphoma; ASCT, autologous stem cell transplantation; CR, complete remission; PR, partial remission; SD, stable disease; PD, progressive disease; ECOG PS, Eastern Cooperative Oncology Group performance status.

**Table S3.** Details of infectious episodes.

|  | **All patients** | **8-day** | **9-day** |
| --- | --- | --- | --- |
| **All infectious events** | **N = 28** | **N = 20** | **N = 8** |
|  |  |  |  |
| **Bacterial infection**  **Gram-positive**  Staphylococcus spp  Streptococcus spp  Enterococcus spp  Clostridioides difficile | **N = 13**  1  2  2  1 | **N = 8**  1  1  1  0 | **N = 5**  0  1  1  1 |
| **Gram-negative**  Escherichia coli  Pseudomonas aeruginosa  Acinetobacter baumannii | 4  1  2 | 2  1  2 | 2  0  0 |
| **Viral infection**  Cytomegalovirus  Herpes simplex virus  Varicella-zoster virus  COVID-19 | **N = 12**  5  4  2  1 | **N = 9**  4  3  1  1 | **N = 3**  1  1  1  0 |
| **Fungal infection**  Pneumocystis jirovecii  Candida spp  Aspergillosis spp  Mucor spp | **N = 3**  1  1  1  0 | **N = 3**  1  1  1  0 | **N = 0** |
|  |  |  |  |
| **Infection site, n (%)**  Bloodstream infection  Catheter-related infection  Lung infection  GI tract infection (including oral mucosa)  Skin infection  Cytomegalovirus reactivation | N = 28  8 (28.6)  4 (14.3)  5 (17.9)  5 (17.9)  2 (7.1)  4 (14.3) | N = 20  5 (25.0)  3 (15.0)  5 (25.0)  3 (15.0)  1 (5.0)  3 (15.0) | N = 8  3 (37.5)  1 (12.5)  0  2 (25.0)  1 (12.5)  1 (12.5) |
|  |  |  |  |
| **Infection onset, n (%)**  During the conditioning period  Pre-engraftment period (D0–30)  Post-engraftment period (> D30) | N = 28  8 (32.0)  15 (60.0)  5 (25.0) | N = 20  3 (15.0)  12 (60.0)  5 (25.0) | N = 8  5 (62.5)  3 (37.5)  0 |

GI, gastrointestinal.

**Table S4.** Individual patient characteristics of those who died from transplantation-related mortality.

|  | **Case 1** | **Case 2** | **Case 3** | **Case 4** | **Case 5** |
| --- | --- | --- | --- | --- | --- |
| **Group** | 9-day | 9-day | 9-day | 9-day | 8-day |
| **Age at ASCT** | 59 | 49 | 58 | 40 | 62 |
| **Sex** | F | M | M | M | F |
| **ECOG PS** | 1 | 1 | 1 | 0 | 1 |
| **HCT-CI** | 2 | 4 | 2 | 1 | 2 |
|  |  |  |  |  |  |
| **Disease**  Histology | PCNSL  IVLBCL | PCNSL  DLBCL | PCNSL  DLBCL | SCNSL  BL | PCNSL  DLBCL |
| **Prior line of Therapy** | 1 | 2 | 1 | 2 | 2 |
|  |  |  |  |  |  |
| **Disease status** | CR1 | PR | PR | CR2 | PR |
|  |  |  |  |  |  |
| **Time from Dx to ASCT** | 5.5 months | 26.0 months | 7.3 months | 11.3 months | 13.7 months |
|  |  |  |  |  |  |
| **Infused CD34** | 4.87 | 5.52 | 4.00 | 6.19 | 2.49 |
|  |  |  |  |  |  |
| **Neutrophil Engraftment**  Time to  engraftment | Yes  10 days | Yes  9 days | Yes  9 days | No  - | Yes  12 days |
| **Platelet Engraftment**  Time to  engraftment | Yes  18 days | No  - | Yes  8 days | No  0 | Yes  16 days |
| **CMV antigenemia** | Yes | No | No | No | Yes |
|  |  |  |  |  |  |
| **OS** | 0.8 months | 0.4 months | 0.9 months | 0.2 months | 1.6 months |
| **COD** | Septic shock | Septic shock | Septic shock | Septic shock | Septic shock |
| **Infection** | Infectious colitis | Pneumonia | Bacteremia | Cellulitis | Pneumonia |
| **Etiology** | *C. difficile* | Not identified | *E. coli* | Not identified | *P. jirovecii* |

ASCT, autologous stem cell transplantation; PS, performance status; HCT-CI, hematopoietic cell transplantation-specific comorbidity index; PCNSL, primary central nervous system lymphoma; SCNSL, secondary central nervous system lymphoma; IVLBCL, intravascular large B-cell lymphoma; DLBCL, diffuse large B-cell lymphoma; BL, Burkitt lymphoma; CR, complete remission; PR, partial remission; Dx, diagnosis; CMV, cytomegalovirus; OS, overall survival; COD, cause of death.

**Table S5.** Transplantation outcomes according to disease type (PCNSL vs. SCNSL).

|  | **PCNSL**  **N = 25** | **SCNSL**  **N = 19** | ***P*-value** |
| --- | --- | --- | --- |
| **Neutrophil engraftment, n (%)**  Time to engraftment, day, median (range) | 25 (100)  11 (9–19) | 18 (94.7)  11 (9–13) | 0.889  0.200 |
| **Platelet engraftment, n (%)**  Time to engraftment, day, median (range) | 24 (96.0)  13 (8–20) | 18 (94.7)  11 (7–24) | 1.000  0.428 |
|  |  |  |  |
| **Remission status after ASCT, n (%)**  CR  PR  SD  PD  Not applicable | 18 (72.0)  1 (4.0)  1 (4.0)  1 (4.0)  4 (16.0) | 14 (73.7)  0  0  4 (21.1)  1 (5.3) | 0.250 |
|  |  |  |  |
| **PFS, median (95% CI)**  1yr PFS rate, % (95% CI)  2yr PFS rate, % (95% CI)  3yr PFS rate, % (95% CI) | 36.5 (4.6–NE)  59.7 (43.2–82.6)  51.2 (34.7–75.5)  51.2 (34.7–75.5) | 32.6 (6.3–NE)  63.2 (44.8–89.0)  56.8 (38.1–84.9)  42.6 (24.2–75.1) | 0.920 |
|  |  |  |  |
| **OS, median (95% CI)**  1yr OS rate, % (95% CI)  2yr OS rate, % (95% CI)  3yr OS rate, % (95% CI) | Not reached (NE–NE)  71.5 (55.7–91.9)  66.8 (50.2–88.7)  66.8 (50.2–88.7) | Not reached (18.2–NE)  73.7 (56.3–96.4)  67.5 (49.1–92.9)  67.5 (49.1–92.9) | 0.800 |
|  |  |  |  |
| **Relapse at 3 years, % (95% CI)** | 38.9 (13.6–56.8) | 55.0 (21.5–74.2) | 0.510 |
| **NRM at 1 year, % (95% CI)** | 16.2 (0.3–29.5) | 5.3 (0–14.8) | 0.300 |

PCNSL, primary central nervous system lymphoma; SCNSL, secondary central nervous system lymphoma; ASCT, autologous stem cell transplantation; CR, complete remission; PR, partial remission; SD, stable disease; PD, progressive disease; CI, confidence interval; NE, not estimable; PFS, progression-free survival; OS, overall survival; NRM, non-relapse mortality.
